# Supplementary material for: Substrate Charge Transfer Induced Ferromagnetism in MnSe/SrTiO3 Ultrathin Films
Source: Nanomaterials (Basel). 2024 Aug 16;14(16):1355. doi: 10.3390/nano14161355 (PMC11356950; doi:10.3390/nano14161355)
Supplement: Supplementary file 1 [file nanomaterials-14-01355-s001.zip › nanomaterials-3097005-supplementary.pdf]

Supplementary information

Substrate charge transfer induced ferromagnetism in $MnSe/SrTiO_3$ ultrathin films

Chun-Hao Huang[1,#], Chandra Shekar Gantepogu[1,2,3,#], Peng-Jen Chen[4], Ting-Hsuan Wu[1,2], Wei-Rein Liu[5], Kung-Hsuan Lin[1], Chi-Liang Chen[5], Ting-Kuo Lee[1,6], Ming-Jye Wang[1,7,*], Maw-Kuen Wu[1]

[1]*Institute of Physics, Academia Sinica, Taipei 11529, Taiwan*

[2]*Department of Physics, National Taiwan University, Taipei 10617, Taiwan*

[3]*Nano Science and Technology Program, Taiwan International Graduate Program, Academia Sinica, Taipei 11529, Taiwan*

[4]*Physics Division, National Center for Theoretical Sciences, Hsinchu 30013, Taiwan*

[5]*National Synchrotron Radiation Research Center, Hsin-Chu 30076, Taiwan*

[6]*Department of Physics, National Sun Yat-sen University, Kaohsiung 80424, Taiwan*

[7]*Institute of Astronomy and Astrophysics, Academia Sinica, Taipei 10617, Taiwan*

[#]*Authors have equal contributions*

[*]*Corresponding authors: mingjye@asiaa.sinica.edu.tw*

I. The optical transmission in MnSe film.

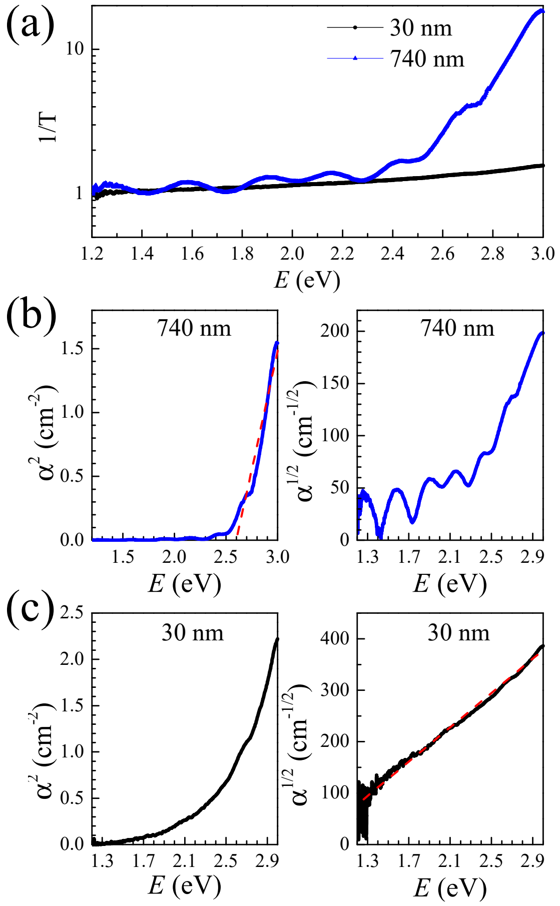

Figure S1 (a) the absorbance spectra of the MnSe films. The spectrum of 740 nm film (blue) shows a rapid increase around 2.6 eV. The oscillation is due to the multi-reflection of photon inside the film. Instead, the spectrum of 30 nm film reveals a gradual increase. The α^2-E plot and $\alpha^{1/2}$-E plot of the (b) 740 nm and (c) 30 nm MnSe films. The dominant electron transition process is through the direct energy gap in 740 nm film and the indirect energy gap in 30 nm film.

The MnSe film with stretched *ab*-plane demonstrates abnormal magnetic and transport property from the semiconducting bulk sample. It is valuable to investigate the change of energy gap by the optical transmission experiments. According to the Bouguer-Lambert-Beer absorption law, the transmittance $T=e^{-\alpha d}$, where α is the absorption coefficient and d is the thickness of the sample. Therefore, the absorbance αd = ln(1/T). Early experiments reported an energy gap near 2 eV in cubic MnSe polycrystalline sample [1, 2]. The theoretical calculations by Youn *et al.* showed a direct energy gap of about 1.3 eV at X-point (<110>) and 2.1 eV at T-point (<111>). The indirect gap between X- and T-points is about 0.63 eV [3]. Later, Amiri *et al.* reported a larger direct energy gap of 1.9 eV at X-point and indirect energy gap of 1.2 eV between T- and X- points [4]. The energy gap at T-point remains a similar value of 2.1 eV. In general, the electron can transit to the conduction band through a direct or indirect gap after absorbing an incident photon. While direct transition is allowed, the absorption

coefficient $\alpha(E)$ is expected to be proportional to $(E\text{-}E_{dGap})^{1/2}$ when the incident photon energy (E) is larger than the direct gap E_{dGap}. For the indirect gap situation, $\alpha(E)$ is proportional to $(E\text{-}E_{iGap})^2$ as $E > E_{iGap}$. Therefore, the α^2-E plot and $\alpha^{1/2}$-E plots can be used to quantitatively determine the direct gap and/or indirect gap, respectively [5].

Fig. S1-(a) shows the absorption spectra of the MnSe films with thickness of 30 nm (black line) and 740 nm (blue line). The absorption spectra of 740 nm film (bulk-like) reveals a dramatic increase near 2.6 eV, which is similar to the absorption spectra of bulk MnSe [1]. It should be noted that the oscillation in the absorption spectrum is due to the multi-reflection inside the MnSe film which usually emerges in thick film sample. In contrast to the 740 nm film, the absorption spectra of 30 nm MnSe monotonically increase with increasing photon energy.

Fig. S1-(b) shows the α^2-E plot (left panel) and $\alpha^{1/2}$-E plot (right panel) of the 740 nm MnSe film. The α^2-E plot reveals a linear behavior at high energy region and an E_{dGap} of 2.6 eV was extracted. The absorption below E_{dGap} is almost negligible. However, the $\alpha^{1/2}$-E plot, which naturally emphasizes the low absorption at low energy region (below 2.4 eV), demonstrates a barely linear energy dependence as the oscillation due to the multi-reflection effect inside the film is ignored. According to the fitting line extrapolated from 2.4 eV to intercept with the energy axis, the indirect gap energy E_{iGaP} is in the range between 0.7 eV and 1.0 eV. The extracted E_{dGap} and E_{iGap} are consistent to the theoretical calculation results [3, 4]. Similarly, the α^2-E and $\alpha^{1/2}$-E plots of the 30 nm MnSe film were shown in the left and right panels of **Fig. S1-(c)**. There is no linear region in α^2-E plot to clearly determine the energy of direct bandgap. On the contrary, the $\alpha^{1/2}$-E plot reveals a linear energy dependence in the measured energy region. The extracted indirect energy gap is around 0.63 eV. From both the $\alpha^{1/2}$-E and α^2-E plots in **Fig. S1-(c)**, they indicate the indirect-gap electron transition is relatively strong in the visible range for the 30 nm ab-plane stretched MnSe film, leading to non-significant feature of direct-gap transition in α^2-E plots. In addition, the minor absorption of the 740 nm film below E_{dGap} can be attributed to the *ab*-plane stretched layer near the substrate.

References.

[1] D. L. Decker, and R. L. Wild, *Phys. Rev. B*, **4**(10), 3425 (1971)
[2] H. Sato *et al.*, *Phys. Rev. B*, **56**(12), 7222 (1997)
[3] S. J. Youn *et al.*, *Phys. status solidi (b)*, **241**(7), 1411-1414 (2004)
[4] P. Amiri *et al.*, *Phys. Rev. B*, **83**(16), 165424 (2011)
[5] A. R. Zanatta., *et al.*, *Sci. rep.*, **9**(1), 1-12 (2019)

II. The 1/ |of 740 nm, 140 nm, and 30 nm MnSe films and susceptiblity of pure STO substrate.

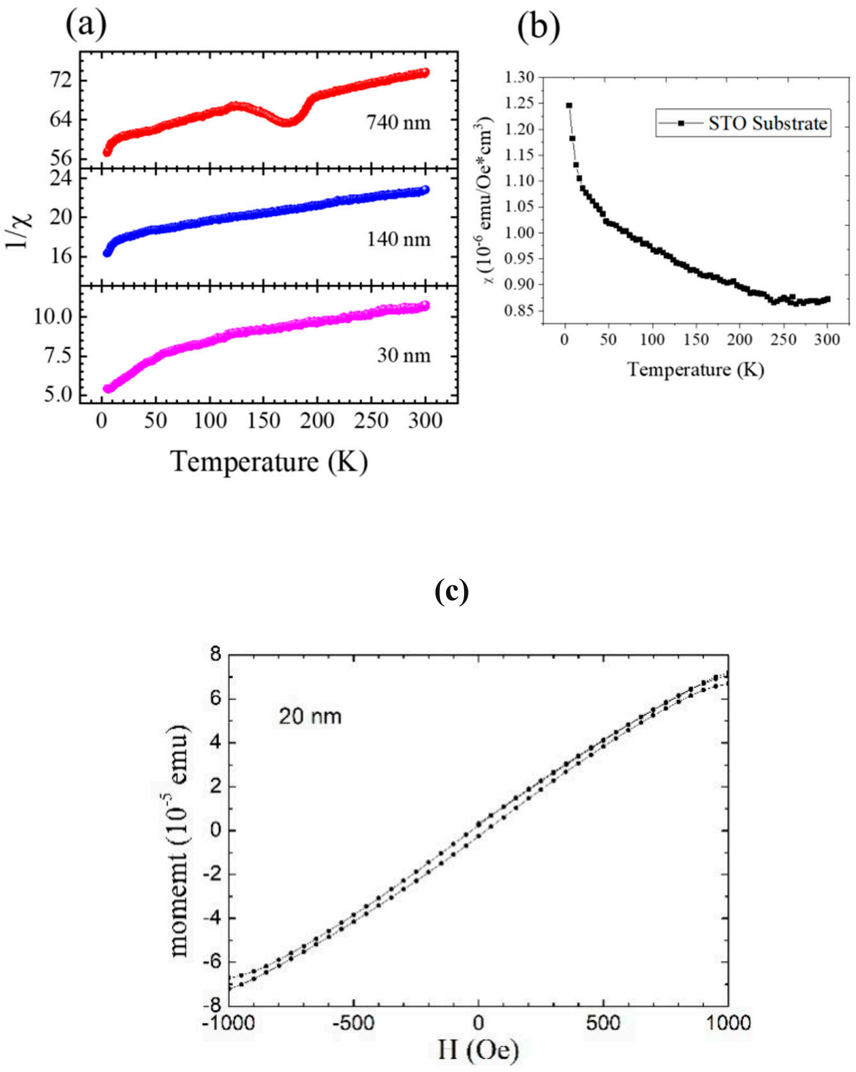

Figure S-2 shows the magnetic data (a) 1/ | vs T for 30 nm, 140 nm, and 740 nm thickness MnSe films and (b) pure STO substrate. (c) The zoom in M-H curve of the 20 nm MnSe film.

III. The temperature dependent M-T and M-H loop of $SrTiO_3$ in comparison with MnSe film.

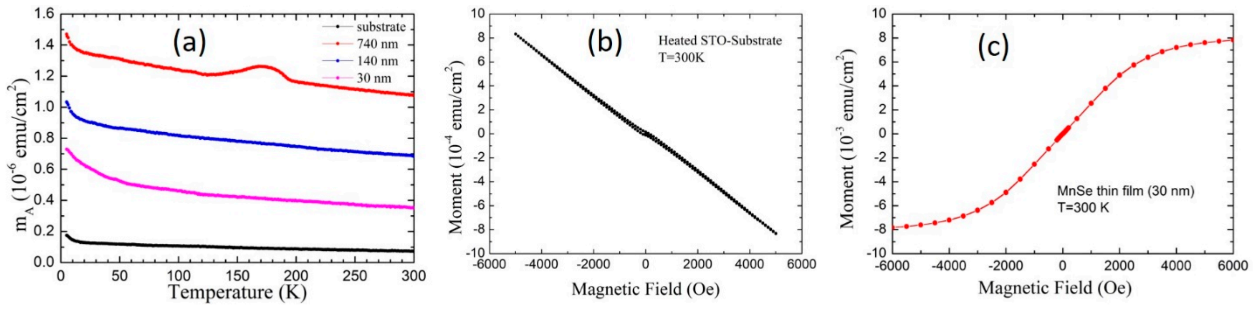

Figure S-3 (a) normalized M-T curves of $SrTiO_3$ substrate and MnSe thin films with different thicknesses. (b) The normalized M-H curve of $SrTiO_3$ substrate. (c) The normalized M-H curve of 30 nm MnSe ultrathin film.

The magnetic signal from the STO substrate was studied. Figure S-3(a) shows the normalized M-T curves of $SrTiO_3$ substrate and MnSe thin films with different thicknesses. The signal of $SrTiO_3$ substrate is much smaller than the samples with MnSe films. Figure S-3 (b) shows the normalized M-H curve of $SrTiO_3$ substrate, which shows a diamagnetic behavior in the fields measured. Figure S-3 (c) shows the normalized M-H curve of 30 nm MnSe ultrathin film, which reveals a superparamagnetic behavior. The data show that the signal of STO substrate is one-order of magnitude smaller.

IV. **The temperature dependent M-H loop of 40 nm MnSe film.**

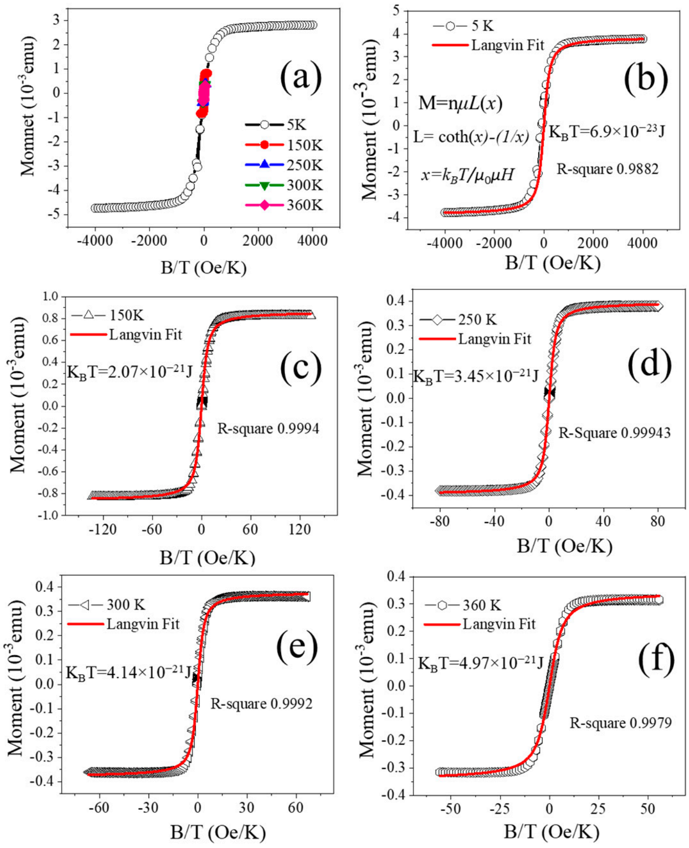

Figure S-4 (a) The temperature dependent M-H curves of 40 nm MnSe film, and each M-H curve (b-f) is fitted using Langevin function, $M=n\mu L(\mu_0\mu H/k_BT)$, where n is the density of superparamagnetic domain, μ is the magnetic moment of each superparamagnetic domain, μ_0 is the magnetic permeability of free space, k_B is the Boltzmann constant, T is the temperature of material, and L is the Langevin function.

V. **The peak fitting of Mn L_3 XANES (X-ray absorption near edge spectrum)**

The spectra of Mn L_3-edge absorption of studied films were analyzed to understand the details of electron transition after absorbing photon. The continuous absorption background, having an arctangent functional form, are subtracted before doing the peak fitting. The Mn L_3-edge absorption spectrum can be well fitted by five peaks with Gaussian profile. The fitted curves (blue, magenta, dark green, wine, and orange lines) and accumulative curve (red dot line) with the experimental data of 740 nm, 140 nm, and 30 nm films are shown in **Fig. S-4(a)**, **Fig. S-4(b)**, and **Fig. S-4(c)** respectively.

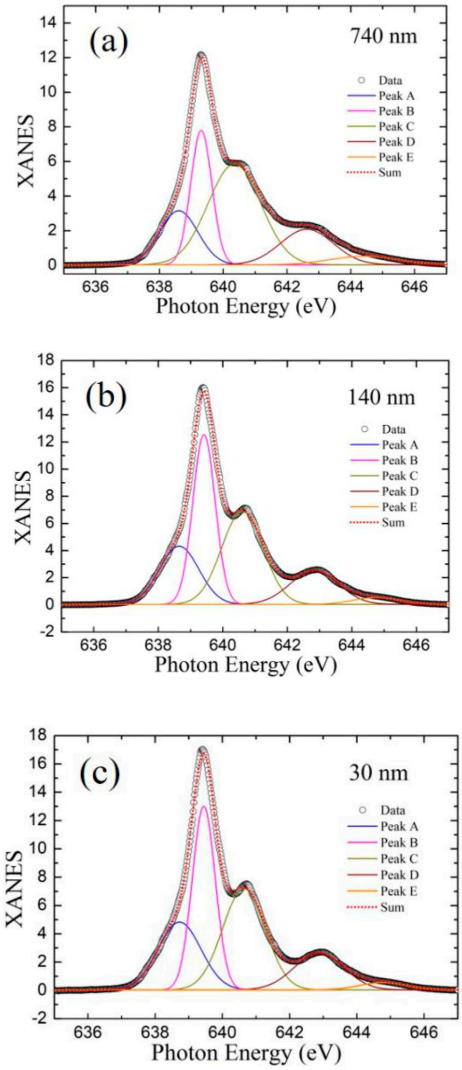

Figure S-5 The peak fitting of Mn L_3-edge absorption spectra of (a) 740 nm, (b) 140 nm, and (c) 30 nm MnSe films. Five peaks with Gaussian profile are used in the fitting. The dot line is the sum of five simulated peaks. The fitting parameters are tabulated in **Table S-1**.

Table S-1 The position, area, and FWHM (full width at half maximum) of the de-convoluted absorption peak of the studied MnSe films. The continuous absorption background with arctangent functional form was subtracted and Gaussian profile was used in the fitting.

		740 nm	140 nm	30 nm
Peak A	Position (eV)	638.603±0.018	638.646±0.022	638.725±0.024
	Area	4.713±0.146	6.557±0.218	7.619±0.272
	FWHM (eV)	1.417±0.022	1.437±0.028	1.495±0.029
Peak B	Position (eV)	639.309±0.001	639.416±0.002	639.445±0.001
	Area	6.461±0.119	10.868±0.217	11.138±0.240
	FWHM (eV)	0.781±0.004	0.816±0.005	0.807±0.005
Peak C	Position (eV)	640.349±0.005	640.605±0.004	640.639±0.004
	Area	12.106±0.205	11.080±0.108	11.361±0.103
	FWHM (eV)	1.954±0.028	1.520±0.015	1.491±0.013
Peak D	Position (eV)	642.623±0.019	642.833±0.011	642.873±0.011
	Area	4.261±0.288	4.674±0.119	4.825±0.110
	FWHM (eV)	1.948±0.062	1.772±0.043	1.752±0.039
Peak E	Position (eV)	644.413±0.174	644.759±0.070	644.783±0.064
	Area	1.315±0.220	0.905±0.092	0.908±0.088
	FWHM (eV)	2.487±0.194	1.688±0.119	1.645±0.113

VI. Charge transfer of MnSe ultrathin film from the SrTiO3 substrate

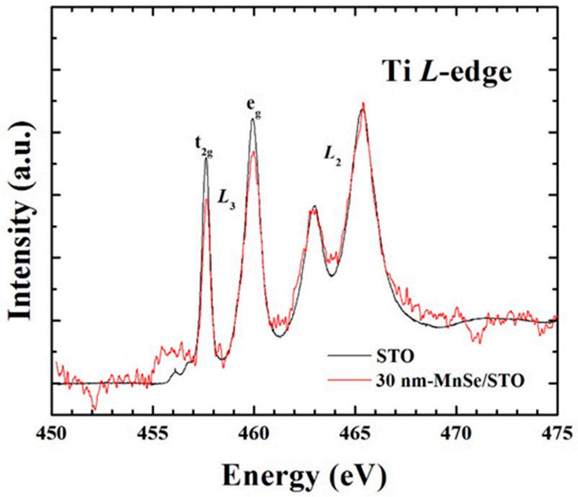

Figure S-6 The Ti L-edge X-ray absorption spectra of 30 nm-MnSe/STO (red line) and pure $SrTiO_3$ subtract films (black line).

The Ti L-edge X-ray absorption spectra of $SrTiO_3$ in both bare substrate (black line) and MnSe on $SrTiO_3$ ultrathin (30 nm) film (red line) were measured using the TEY (total electron yield) mode at the TLS BL20A beamline of NSRRC Taiwan, as shown in **Fig. S-6**. The peaks in the spectra can be identified as the L_2- and L_3- edge absorptions. The L_3 edge contains two components as marked e_g and t_{2g}. The shape and center of e_g and t_{2g} peaks in both samples are almost identical which reveals the similar coordination environment of Ti.

The spectra clearly show that the unoccupied state number of L_3 in $SrTiO_3$ is higher than that in 30 nm-MnSe/STO. This result indicates that some electrons are transferred from MnSe film to the $SrTiO_3$ substrate, and equivalently some holes are transferred from to the $SrTiO_3$ substrate to the MnSe film, near the interface. The extra holes could transfer the magnetic property of MnSe from A-type AFM to FM which is an energetically favored state, as discussed in the theoretical study part of the main text. This FM nano-grains near the interface cause the emergence of superparamagnetism.

VII. The atom arrangement of the MnSe(100)/$SrTiO_3$(110)

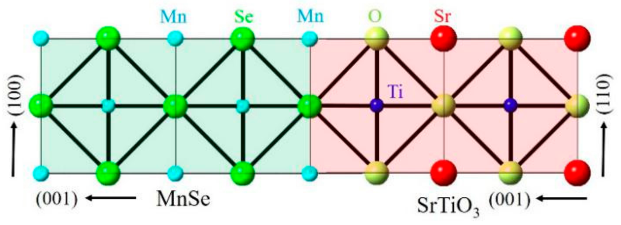

Figure S-7 The atom arrangement along the c-axis of the MnSe (100) on $SrTiO_3$ (110)

Figure S-7 is the atom arrangement along the c-axis of the MnSe (100) on $SrTiO_3$ (110). Such arrangement is similar to to that near the interface of the ABO_3/$AB'O_3$ perovskites, the Fig. 1(b) in [1]. The electron of Mn could transfer to the Ti^{4+} site of the $SrTiO_3$ due to the same charge transfer mechanism.

[1] Z. Zhong et al. *Phys. Rev. X* **7**, 011023 (2017)

VIII. Carrier type in MnSe film.

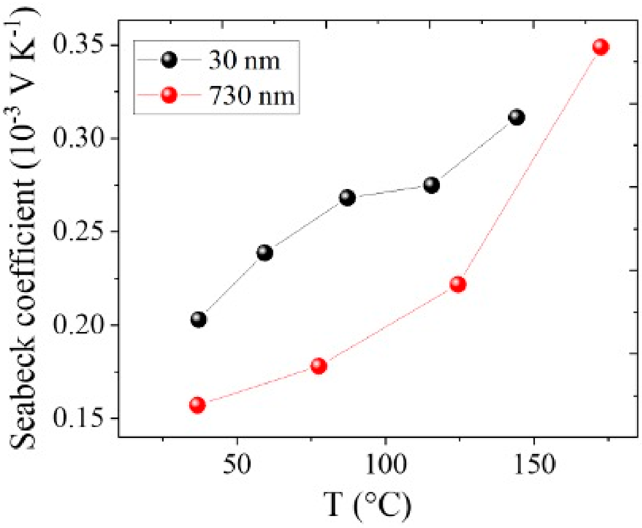

Figure S-8 Seebeck coefficient versus the temperature of the low and high thick samples.

Indeed, the carrier should be p-type according to our XAS results. The unoccupied state of Mn L-edge in MnSe/STO is enhanced as the thickness increased and that of Ti L-edge decreases in comparison with pure STO. This observation indicates hole doping from STO into MnSe. In addition, we add a

reference in the main text, Zheng, Liangtao, et al. "Thermoelectric properties of p-type MnSe." *Journal of Alloys and Compounds* 789 (2019): 953-959 to support our claim [1]. Unfortunately, the Hall effect measurements cannot be carried out in our PPMS system because of the high resistance of the samples. Instead, we conducted Seebeck measurements above room temperature. The results clearly show the presence of p-type carriers for 30 nm and 730 nm films as shown in the following graph. We have added this information to the supplementary materials. Furthermore, we estimate the carrier concentration based on $S=(8\pi^2(k_B)^2T/(2eh^2))m^*(\pi/3n)^{2/3}$ formula, where k_B is Boltzmann constant, T is temperature; h is Planck constant; S is Seebeck coefficient (measured data: $S=2\times10^{-4}$ V/K at 300 K); m* is effective mass; e:elementary charge; n is carrier concentration. The estimated carrier concentration is $n=2.07\times10^{18}$ cm^{-3}.

[1] Zheng, Liangtao *et al.*, *J. Alloys Compd.* 789 (2019): 953-959.
